# Differentiation and cell density upregulate cytochrome *c* levels in megakaryoblastic cell lines: Implications for analysis of *CYCS*-associated thrombocytopenia

**DOI:** 10.1371/journal.pone.0190433

**Published:** 2017-12-29

**Authors:** Lily Ong, Kirstin O. McDonald, Elizabeth C. Ledgerwood

**Affiliations:** Department of Biochemistry, School of Biomedical Sciences, University of Otago, Dunedin, New Zealand; Katholieke Universiteit Leuven, BELGIUM

## Abstract

Mutations in the cytochrome *c* gene (*CYCS*) cause autosomal dominant thrombocytopenia by an unknown mechanism. While attempting to generate megakaryoblastic cell lines exogenously expressing cytochrome *c* variants, we discovered that endogenous cytochrome *c* expression increased both upon induction of differentiation with the phorbol ester phorbol 12-myristate 13-acetate (PMA), and as cell density increased. A concomitant increase in cytochrome *c* oxidase subunit II in response to PMA, but not cell higher cell density, suggests upregulation of the mitochondrial respiratory chain may be a specific feature of differentiation. These results highlight the likely importance of cytochrome *c* in both differentiating and proliferating cells, and illustrate the unsuitability of megakaryoblastic lines for modeling *CYCS*-associated thrombocytopenia.

## Introduction

Cytochrome *c* is a _~_12 kDa heme protein localized to the mitochondrial intermembrane space. Cytochrome *c* plays a pivotal role in various cellular processes [1]. It is an integral component of the mitochondrial respiration machinery where it transfers electrons between complexes III and IV. Cytochrome *c* is essential in the intrinsic apoptosis pathway where it is required for apoptosome assembly and subsequent caspase activation [2,3]. Cytochrome *c* also has peroxidase activity, which is proposed to catalyze cardiolipin oxidation, leading to release of cytochrome *c* and other proapoptotic factors from the mitochondria into cytosol during the early phase of apoptosis [4].

Mutation of the *CYCS* gene causes mild autosomal dominant thrombocytopenia (OMIM 612004, THC4) characterized by a decreased number of functionally normal circulating platelets. To date, three independent *CYCS*-associated cases have been reported. The first case was described in a large New Zealand family (Thrombocytopenia Cargeeg, c.132G>A; p.Gly42Ser) [5], the second case in an Italian family (c.145T>C; p.Tyr49His) [6], and the third case in a United Kingdom cohort (c.155C>T; p.Ala52Val) [7].

Studies of G41S and Y48H cytochromes *c* (numbering based on the mature protein lacking the initiating Met residue) indicate that these mutations enhance the ability of cytochrome *c* to activate caspases *in vitro* [5,6]. However there is no obvious impact of the G41S mutation on induction of apoptosis in peripheral blood mononuclear cells from affected subjects [8]. There is conflicting data on the impact of the G41S and Y48H mutations on mitochondrial respiration *in vitro* with no impact reported for G41S cytochrome *c* [5] but a decreased ability to support O_2_ consumption reported for Y48H cytochrome *c* [6]. In addition the G41S mutation has been shown to increase the peroxidase activity of cytochrome *c*, but does not alter the kinetics of cytochrome *c* release from mitochondria in response to an apoptotic stimulus [9].

Platelets are derived from megakaryocytes, a rare large nucleated cell that resides primarily in the bone marrow, and are released into the bloodstream via long thin, intravascular protrusions termed proplatelets [10]. Analysis of bone marrow and CD45^+^-derived megakaryocytes from Thrombocytopenia Cargeeg subjects has demonstrated that the G41S cytochrome *c* mutation causes an abnormal process of platelet release in the bone marrow, and a possible enhancement of megakaryocyte differentiation/maturation [8]. However the underlying molecular basis of *CYCS*-associated thrombocytopenia and the specific role of cytochrome *c* in platelet formation are unknown.

As there are limitations in the analyses that can be performed on patient-derived cells, numerous studies of megakaryocyte biology have been performed using cell lines with megakaryocyte-like features, including the ability to produce platelets. Here we aimed to investigate the role of cytochrome *c* in megakaryocyte maturation and platelet production in greater detail by overexpressing cytochrome *c* variants in the megakaryoblastic cell line SET-2 [11], thus mimicking the heterozygous presence of the mutation in human subjects. In doing so we uncovered an unexpected upregulation of levels of endogenous cytochrome *c* upon SET-2 differentiation. Additionally, endogenous cytochrome *c* levels increased as cell density increased. From these results we conclude that megakaryoblastic cell lines are unsuitable for modeling of *CYCS*-associated autosomal dominant thrombocytopenia, and propose that increased expression of cytochrome *c* is a feature of megakaryocyte maturation.

## Materials and methods

### Cloning

pcDNA3 vectors (Invitrogen) encoding human WT or G41S cytochrome *c* were constructed by PCR-amplification of the cytochrome *c* coding sequence from pBTR (hCc) or pBTR (hG41S Cc) [5] using the primers BamHI cyt c 5’ (TATTCAGGATCCATG GGCGACGT) and EcoRI cyt c 3’ (TACCAGGAATTCTCATTCGTTCGT). The resulting PCR fragments were then digested with *Bam*HI and *Eco*RI, and inserted into the *Bam*HI/*Eco*RI sites of the pcDNA3 vector. The constructs were confirmed by DNA sequencing.

### Cell culture

SET-2 (DSMZ ACC 608, a kind gift from Dr Maggie Kalev, University of Auckland, New Zealand [12]), MEG-01 (ATCC CRL-2021) and U937 (ATCC CRL-1593.2) cells were cultured in RPMI 1640 (Gibco) supplemented with fetal bovine serum (10%), L-glutamine (2 mM), sodium bicarbonate (2 g/L), penicillin (100 U/mL) and streptomycin (100 μg/mL) at 37°C in a 5% CO_2_ humidified atmosphere. HeLa (ATCC CCL-2) cells were cultured in DMEM (Gibco) supplemented with fetal bovine serum (10%), L-glutamine (4 mM), sodium bicarbonate (3.7 g/L), glucose (3.5 g/L), penicillin (100 U/mL) and streptomycin (100 μg/mL) at 37°C in a 5% CO_2_ humidified atmosphere. Phorbol 12-myristate 13-acetate (PMA) was from Sigma-Aldrich.

Prior to electroporation using the Neon^®^ Transfection System (Invitrogen), pcDNA3, pcDNA3-hWT and pcDNA3-hG41S vectors were linearized by digestion with ScaI (Roche). SET-2 cells were washed twice in Dulbecco’s PBS and then suspended in Resuspension Buffer R at 1 × 10^7^ cells/mL. The cell suspension (100 μL) was mixed with plasmid DNA (2.8 μg) and electroporation carried out at 1400 V with 20 ms pulse width for 2 pulses using the 100 μL Neon^®^ Tips. Stably transfected cells were selected by treatment with 500 μg/mL G418 (Gibco) for 2 to 3 weeks with the media replenished every 2 to 3 days. Single cell clones were isolated by plating the cells at limiting dilution in a 96-well plate. After 2 weeks single colonies were expanded. Single cell clones were screened for overexpression of cytochrome *c* by western blotting.

The IncuCyte Live-Cell Imaging system was used to analyse cell proliferation. Images (16 fields/well) were acquired every 2 h for 120 h. Data were analysed using the IncuCyte Confluence software version 1.5.

### Western blotting

SET-2, MEG-01 and U937 cells were lysed in Triton X-100 lysis buffer (120 mM KCl, 1% Triton X-100, cOmplete^™^ Mini EDTA-free Protease Inhibitor Cocktail (Roche) in Dulbecco’s PBS). HeLa cells were lysed in RIPA buffer (10 mM Tris pH 7.5, 150 mM NaCl, 1% Nonidet P-40, 1% sodium deoxycholate, 0.1% SDS, cOmplete^™^ Mini EDTA-free Protease Inhibitor Cocktail). Following 30 min incubation on ice, the lysates were clarified by centrifugation at 15,000 *g* for 20 min at 4°C and stored at -80°C. Protein concentrations were quantified using a BCA assay. Cell lysates (20 μg total protein) were resolved by 15% reducing SDS-PAGE then transferred to nitrocellulose membrane (0.2 μm, Bio-Rad) and blocked with SEA BLOCK blocking buffer (1:10; Thermo Fisher Scientific). The membrane was incubated overnight at 4°C with anti-cytochrome *c* [7H8.2C12] (1:2,000; BD Biosciences 556433) and anti-MTCO2 (1:1,000; Abcam ab110258) in TBST. Following incubation with anti-mouse IgG HRP conjugate (1:10,000; Bio-Rad 170–6516) blots were developed with SuperSignal West Pico chemiluminescent substrate (Thermo Fisher Scientific). To reprobe the membrane, the membrane was incubated in stripping buffer (0.2 M glycine, 0.1% SDS, 1% Tween20, pH 2.2) at room temperature for 10 min, washed for 2 × 10 min in PBS and 2 × 5 min in TBST before re-blocking with SEA BLOCK blocking buffer. The membrane was incubated overnight at 4°C with anti-Prdx3 (1:10,000; Abcam ab15573) and anti-actin (1:2,000; Sigma-Aldrich A5060) in TBST. Following incubation with anti-rabbit IgG HRP conjugate (1:20,000; Bio-Rad 170–6515) membranes was then developed as above. Densitometry analysis was conducted in the manual work flow of the ImageQuant software package (GE Healthcare). Bands were detected by the minimum slope method, while background subtraction was achieved with the rolling-ball algorithm of this software. Raw data was exported and expressed as a ratio of control signal.

### Flow cytometry

SET-2 or MEG-01 cells were harvested, washed with Dulbecco’s PBS, resuspended in 100 μL of Dulbecco’s PBS and stained with anti-CD41a V450 (1:20; BD Biosciences 561425) and anti-CD61 PE (1:20; BD Biosciences 555754) for 30 min in the dark at RT. After incubation, the cells were washed with Dulbecco’s PBS, resuspended in 400 to 500 μL of FACS buffer (0.01% sodium azide, 0.1% BSA in Dulbecco’s PBS) and analysed on a BD LSRFortessa^™^ cell analyzer (BD Biosciences). Data were analysed using FlowJo Version 10. V450 IgG1 (1:20; BD Biosciences 560373) and PE IgG1 (1:20; BD Biosciences 555749) were used as isotype controls. Platelet-like particles were gated using FSC and SSC parameters determined from platelets isolated from human peripheral blood [13].

### qPCR

Total RNA was isolated from up to 1 × 10^7^ cells using the RNeasy mini kit (Qiagen) with on column DNAse I digestion according to the manufacturer’s instructions and cDNA synthesis was performed from 0.2 μg of total RNA with random primers using the High-Capacity cDNA Reverse Transcription Kits with RNase inhibitor (Applied Biosystems) according to the manufacturer’s instructions. Real-time-PCR was carried out on the LightCycler^®^ 480 Instrument II (Roche). The 10 μL PCR mix contained 2 μL of cDNA template (diluted 1:100), 0.4 μL of forward and reverse primer mix (0.2 μM per primer), 5 μL of 2× KAPA SYBR^®^ FAST qPCR Master Mix Universal (Kapa Biosystems) and 2.6 μL of nuclease-free H_2_O. The PCR conditions were: one cycle at 95°C for 5 min, 45 cycles of amplification at 95°C for 5 s, 60°C for 20 s and 72°C for 8 s. Subsequently, a dissociation program was applied with one cycle at 95°C for 30 s, 65°C for 5 s and heating with a ramp rate of 0.11°C/s to 95°C. A melt curve was produced to confirm a single gene-specific peak and to detect primer-dimer formation. The resulting real-time PCR products were run on a 2% agarose gel to confirm the absence of nonspecific amplification and primer-dimer formation. All reactions were performed in triplicate, with minus reverse transcriptase and H_2_O controls. Selection of the most stable reference genes was performed using NormFinder [14]. Quantification cycle (Cq) was calculated by the LightCycler^®^ 480 software version 1.5 and converted into a fold change relative to reference genes *RPL27* and *HPRT*. The final values were normalized by the control group.

## Results

### PMA induces expression of endogenous cytochrome *c* in SET-2 cells

**Fig 1:**
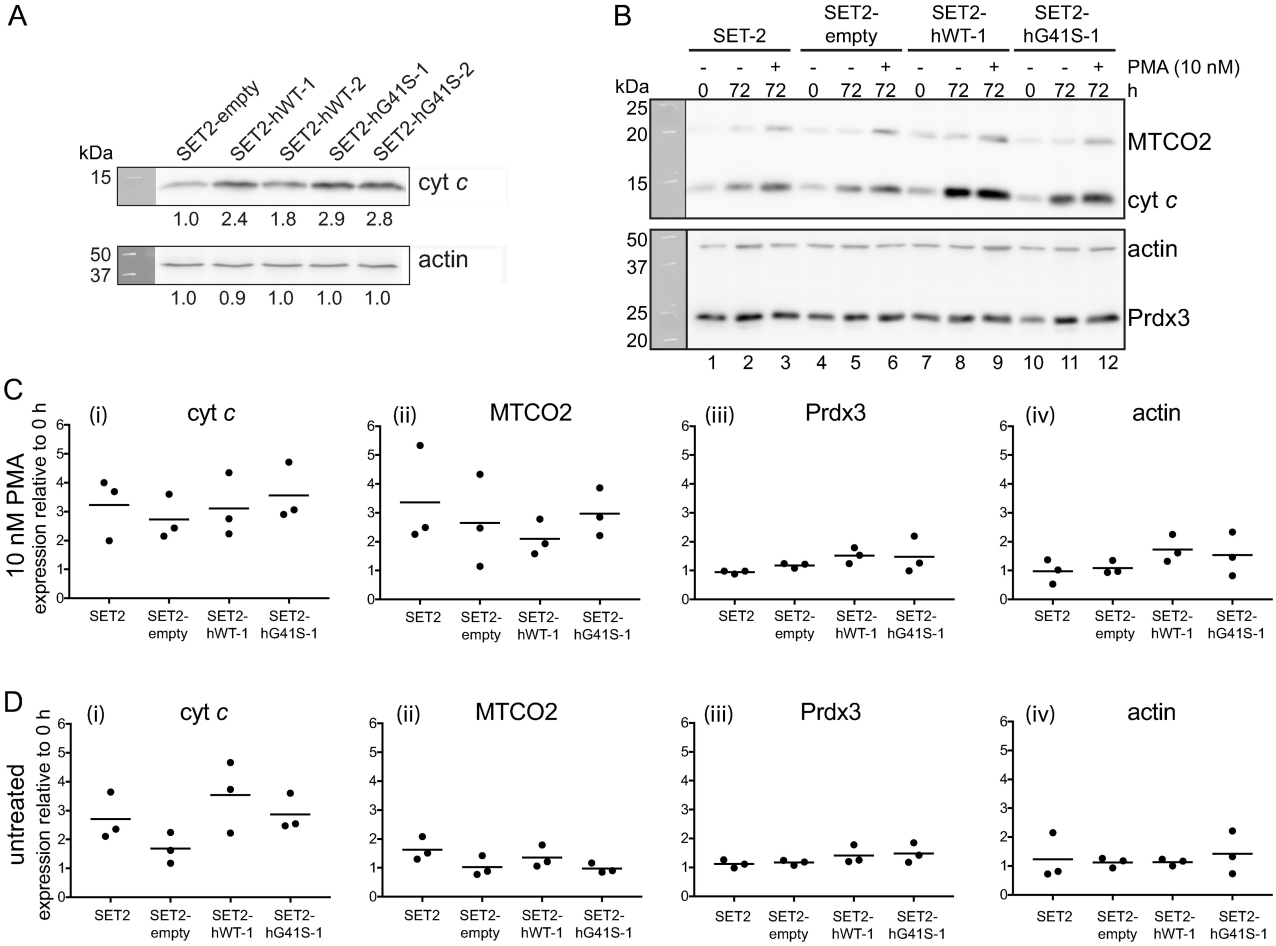
Cytochrome *c* expression in SET-2 cells. **A**. Western blot analysis of cell lysate (20 μg) prepared from SET-2 clonal lines stably transfected with pcDNA3 (SET2-empty), pcDNA3-hWT (SET2-hWT-1 & -2) or pcDNA3-hG41S (SET2-hG41S-1 & -2) probed with anti-cytochrome *c* (upper panel) or anti-actin (lower panel). Numbers below each lane are fold change in expression compared to SET2-empty. **B–D**. SET-2 cells were plated at 2 x 10^5^ cells/mL, treated with or without 10 nM PMA for 72 h, and whole cell lysates (20 μg) analysed for the expression of cyt *c*, MTCO2, Prdx3 and actin. The western blot shown (**B**) is representative of three independent experiments. Fold change in expression of cyt *c* (i), MTCO2 (ii), Prdx3 (iii) and actin (iv) relative to 0 h in PMA treated (**C**) or untreated (**D**) cells. Results presented as individual values with mean, n = 3.

In order to investigate the impact of heterozygous expression of mutant cytochromes *c* on megakaryocyte maturation and platelet formation we attempted to establish SET-2 cells overexpressing either WT or G41S cytochrome *c*. We initially isolated clones with apparent 2–3 –fold overexpression of WT (SET2-hWT-1 & -2) or G41S (SET2-hG41S-1 & -2) cytochrome *c* compared to the cells stably transfected with the parental pcDNA3 vector (SET2-empty) (Fig 1A). Since PMA has been reported to stimulate the activity of the cytomegalovirus promoter in pcDNA3 [15–17] we examined the effect of PMA on cytochrome *c* expression in untransfected and stably transfected SET-2 cells. We first confirmed that PMA treatment induced SET-2 differentiation characterized by inhibition of cell growth (S1A Fig), increased expression of megakaryocyte-specific cell surface proteins CD61 and CD41a (S1B Fig), and increased expression of CD41a/CD61 on released platelet-like particles (S1C Fig). We next determined whether PMA treatment altered the expression of the exogenous cytochrome *c* (Fig 1B–1D). This led to three unexpected observations. Firstly at 0 h cytochrome *c* levels in the SET2-hWT-1 and SET2-hG41S-1 clonal cell lines were similar to untransfected and SET2-empty cells (Fig 1B, compare lanes 1, 4, 7 and 10). This suggested that the clonal lines no longer expressed the exogenous cytochrome *c*. Secondly in response to PMA-induced differentiation cytochrome *c* expression increased in all cell lines (Fig 1B & 1Ci). Thirdly cytochrome *c* expression increased after 72 h in culture without PMA treatment (Fig 1B & 1Di). Taken together these observations suggested that endogenous cytochrome *c* is upregulated both as SET-2 cells differentiate and as cell density increases, and that the “overexpressing” clonal lines originally identified (Fig 1A) may have been an artefact of differences in cell density at the time of analysis.

The increase in the level of cytochrome *c* in both PMA treated and untreated cells could be due to either general mitochondrial biogenesis or a specific increase of the mitochondrial respiratory chain complexes. We therefore analysed the expression of two other mitochondrial proteins, peroxiredoxin 3 (Prdx3) and cytochrome *c* oxidase subunit II (MTCO2), and compared their expression with cytosolic actin. Prdx3 is a nuclear-encoded mitochondrial matrix protein [18] and MTCO2 is mitochondrial-encoded subunit of cytochrome *c* oxidase [19]. Similar to cytochrome *c*, 72 h PMA treatment induced an average 2.8-fold increase in MTCO2 expression across all cell lines (Fig 1Cii, Table 1). In contrast the expression of Prdx3 (1.3-fold) remained consistent with the variation in actin expression (1.3 fold) (Fig 1Ciii and 1Civ, Table 1). This suggests that there may be specific upregulation of mitochondrial respiratory chain complexes during PMA-induced differentiation of SET-2 cells. In untreated cells there was little change in expression of MTCO2 or Prdx3 (Fig 1Diii and 1Div, Table 1) suggesting that the cell proliferation-induced increase in cytochrome *c* expression is not related to mitochondrial biogenesis or upregulation of respiratory chain complexes.

**Table 1:** Changes in expression of cyt *c*, MTCO2, Prdx3 and actin in untreated and PMA treated Set-2 cells.

	average fold change in protein expression after 72 h (± SD, n = 12^a^)			
	cyt *c*	MTCO2	Prdx3	actin
+ PMA	3.2 ± 0.9	2.8 ± 1.2	1.3 ± 0.4	1.3 ± 0.5
- PMA	2.7 ± 1.0	1.2 ± 0.4	1.3 ± 0.3	1.2 ± 0.5

^a^combined data from all cell lines

### Upregulation of cytochrome *c* expression is a general feature of PMA-induced differentiation

**Fig 2:**
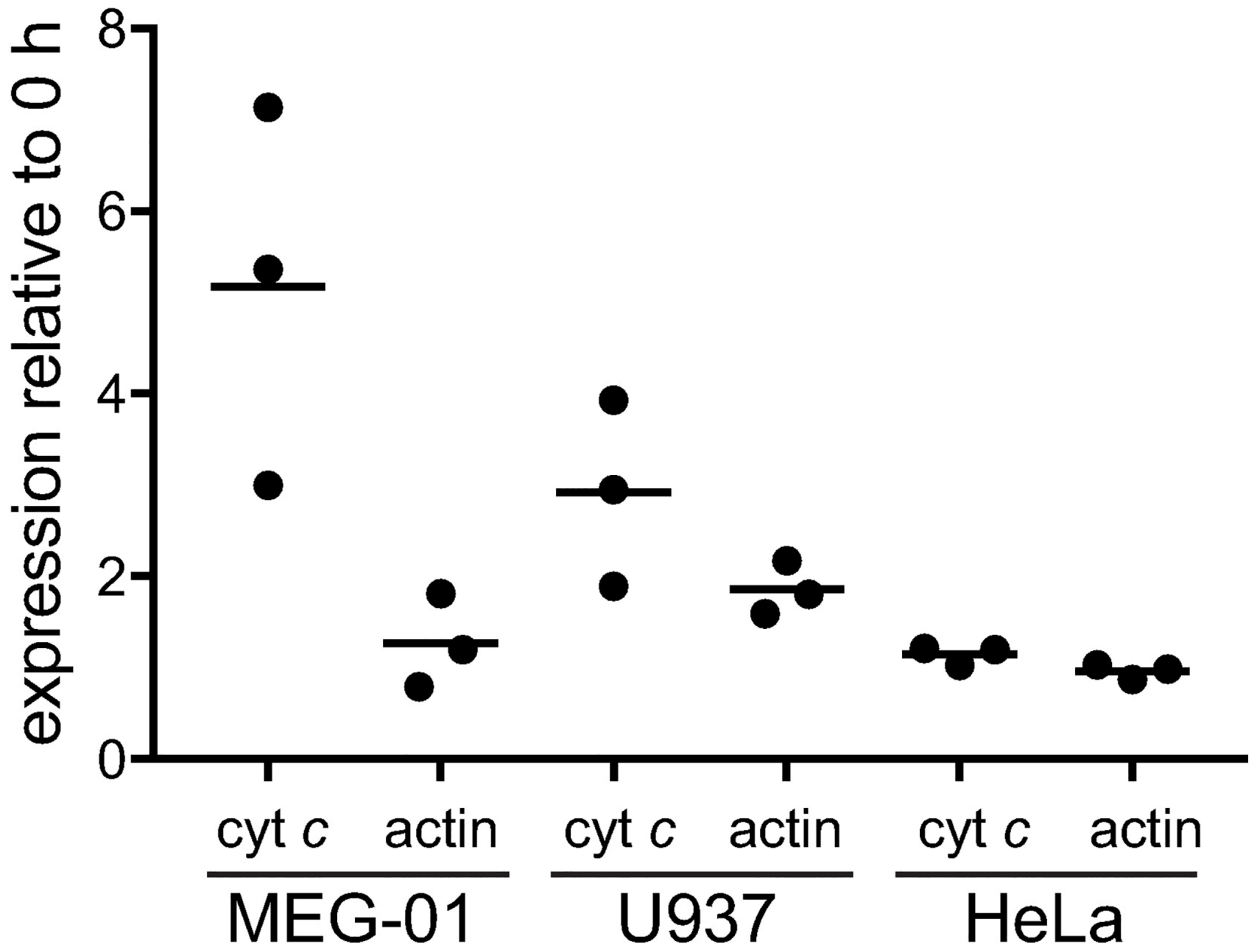
Effect of PMA on cytochrome *c* expression in MEG-01, U937 and HeLa cells. Cells plated at 2 x 10^5^ cells/mL, incubated in the presence of 5 nM (MEG-01) or 10 nM (U937, HeLa) PMA for 72 h. Whole cell lysates (20 μg) analysed by western blot for expression of cyt *c* and actin. Fold change in expression relative to 0 h. Results presented as individual values with mean, n = 3.

In SET-2 cells, treatment with PMA induced both differentiation and increased expression of cytochrome *c*. As PMA is known to induce differentiation of a range of cells we investigated whether PMA-induced upregulation of cytochrome *c* was specific to megakaryopoiesis or a general feature of PMA-induced differentiation. We analysed a second megakaryocytic cell line, MEG-01 [20], the U937 monocytic cell line in which PMA induces growth arrest and differentiation into adherent macrophage-like cells [21,22], and HeLa cells, which do not undergo differentiation (Fig 2). Treatment with PMA induced MEG-01 cell differentiation (S2 Fig) and resulted in a 3–7 fold increase in cytochrome *c* expression, similar to that observed in SET-2 cells. PMA-treated U937 cells adhered to culture dishes, an indication of full differentiation into macrophages, and there was a 2–4 fold increase in cytochrome *c* expression after 72 h. The expression level of cytochrome *c* and actin remained unchanged in PMA-treated HeLa cells.

The differentiation of hematopoietic stem cells towards the megakaryocyte lineage is driven by thrombopoietin (TPO), which signals via the c-Mpl receptor [23]. PMA is a pharmacological differentiation-inducing agent and triggered upregulation of cytochrome *c* expression in U937, SET-2 and MEG-01 cells. We next attempted to investigate whether cytochrome *c* levels increased during TPO-induced megakaryocyte differentiation of peripheral blood mononuclear cell-derived hematopoietic stem cells [8]. As the limited number of peripheral blood cells isolated from a single experiment (15–30 × 10^6^ cells/35 mL of blood) hampered our attempt to analyze cytochrome *c* expression by western blotting, we turned to analysis of mRNA. However cytochrome *c* mRNA levels slightly decreased in PMA-treated SET-2 cells (S3 Fig). Thus the observed change in cytochrome *c* expression occurs at a post-transcriptional level. As an alternative approach, we attempted to measure cytochrome *c* levels by flow cytometry of permeabilized SET-2 cells stained with an anti-cytochrome *c* antibody. This was unsuccessful due to high background staining from the secondary antibody. Alternative approaches will be required to determine whether increased cytochrome *c* expression is a feature of TPO-induced megakaryopoiesis.

### Cytochrome *c* expression varies with cell density

**Fig 3:**
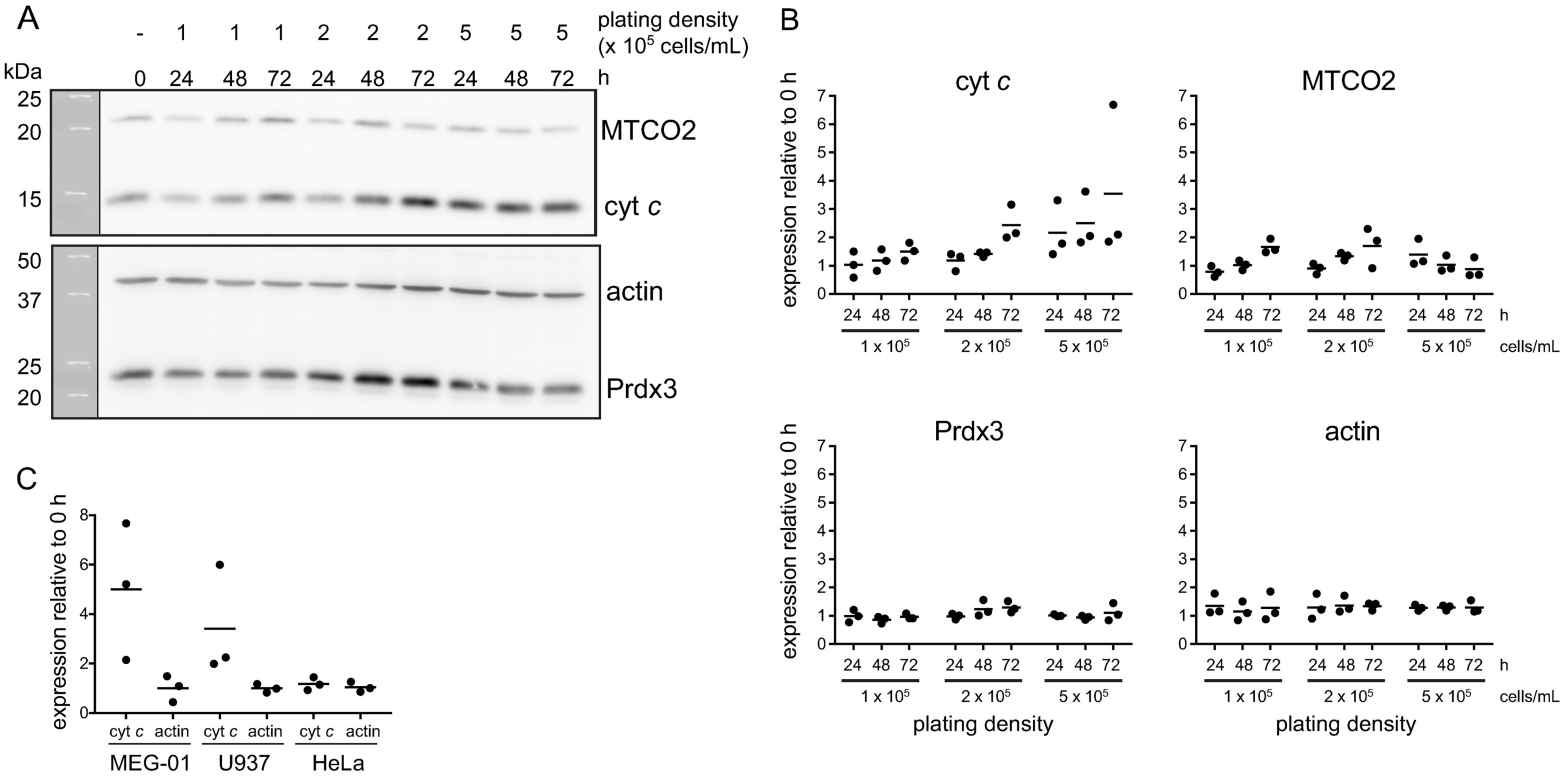
Cytochrome *c* expression varies with cell density. **A & B**. SET-2 cells were plated at 1, 2 or 5 x 10^5^ cells/mL and cultured for 72 h. **A**. Whole cell lysates (20 μg) analysed by western blot for the expression of cytochrome *c*, MTCO2, Prdx3 and actin. The western blot shown is representative of three independent experiments. **B**. Fold change in expression of cytochrome *c*, MTCO2, Prdx3 and actin relative to 0 h. **C**. MEG-01, U937 or HeLa cells were plated at 2 x 10^5^ cells/mL, cultured for 72 h, and whole cell lysate (20 μg) analysed by western blot for expression of cyt *c* and actin. Fold change in expression relative to 0 h. Results presented as individual values with mean, n = 3.

The expression of certain genes is dependent on cell density [24–27]. Having observed an increase in cytochrome *c* expression after 72 h growth (Fig 1D) we hypothesized that that the “overexpressing” clonal lines originally identified (Fig 1A) were an artefact of differences in cell density at the time of analysis and that cell density has an impact on endogenous cytochrome *c* expression. To test this hypothesis, SET-2 cells were plated at three different densities (1, 2 and 5 × 10^5^ cells/mL) and the expression of cytochrome *c*, MTCO2, Prdx3 and actin analysed after 24, 48 and 72 h (Fig 3A & 3B). The analysis revealed that expression of endogenous cytochrome *c* tended to increase as cell density increased and was quite variable, whereas there was little change in the expression of MTCO2, Prdx3 and actin (Fig 3B). A similar result was seen in MEG-01 and U937 cells where cytochrome *c* but not actin expression tended to increase over 72 h (Fig 3C). In contrast cell proliferation had no impact on cytochrome *c* expression in HeLa cells (Fig 3C). This result strongly supports the hypothesis that the apparent expression of exogenous WT or G41S cytochrome *c* in SET2-hWT-1&2 and SET2-hG41S-1&2 (Fig 1A) was most likely due to harvesting cells at varying density rather than overexpression from the pcDNA3 vector. It is not known why no true overexpressing cell lines were identified.

## Discussion

Heterozygous expression of mutant cytochromes *c* causes autosomal dominant thrombocytopenia characterized by alterations in megakaryopoiesis and platelet release [5–8]. To investigate the basis of this phenotype we aimed to recapitulate the heterozygous expression of mutant cytochromes *c* in megakaryoblastic cell lines. The degree of cytochrome *c* overexpression in these clonal lines was inconsistent. This led to our discovery that the level of endogenous cytochrome *c* increased in response to both PMA-induced differentiation and higher cell density. This meant it was not possible to isolate clonal megakaryocytic cell lines with stable overexpression of mutant cytochromes *c* for investigation of the underlying biology of *CYCS*-associated thrombocytopenia. In future alternative methods such as the use of either patient-derived or CRISPR-Cas9 modified iPS cells should be used for these investigations.

The variation in cytochrome *c* levels in SET-2, MEG-01 and U937 cells in response to both PMA-induced differentiation and increasing cell density was unexpected. Cytochrome *c* is generally considered a house keeping protein with a relatively long half-life (5–6 days in multiple rat tissues [28], 7.3 days in platelets [29]). Despite this, several studies report regulation of cytochrome *c* by both synthesis and degradation [30–33]. The control of cytochrome *c* expression at the transcriptional level has been extensively studied and is coordinated with other components of the mitochondrial respiratory chain complexes to meet the energy demands of different cells and tissues [34]. However relatively little is known about the post-transcriptional regulation of cytochrome *c* [35] although RNA binding proteins that either suppress or promote cytochrome *c* translation have been described [36].

Cytochrome *c* expression increased in SET-2, MEG-01 and U937 cells but not HeLa cells as cell density increased. While high cell density has been reported to induce neuroendocrine transdifferentiation of prostate cancer cells, osteogenic and chondrogenic differentiation of human mesenchymal stem cells, and keratinocyte differentiation [37–40], expression of the differentiation markers CD41 and CD61 on SET-2 or MEG-01 cells did not increase as cell density increased. The upregulation of cytochrome *c* levels is unlikely to be due to a general increase in the levels of the mitochondrial respiratory chain complexes associated with mitochondrial biogenesis as there was no change in the expression of cytochrome *c* oxidase subunit II or peroxiredoxin 3. Interestingly, cytochrome *c* expression has been reported to increase in response to serum-stimulated growth via activation of CREB [41], a transcription factor for cytochrome *c* but not other nuclear-encoded mitochondrial respiratory chain proteins [34].

Our results are consistent with other reports suggesting upregulation of components of the respiratory chain is a feature of megakaryopoiesis. Mitochondrial biogenesis has been observed during TPO-induced differentiation of CD34^+^ hematopoietic stem cells [42], PMA-induced differentiation of K562 leukemia cells [43] and LiCl-induced differentiation of DAMI megakaryoblastic cells [44]. There was no change in cytochrome *c* mRNA in differentiating CD34^+^ cells [42], consistent with our observation that cytochrome *c* protein but not mRNA increases upon PMA-stimulated differentiation of SET-2 cells. The regulation of expression of mitochondrial-encoded components of the respiratory chain is post-transcriptional [45]. Therefore the increase in cytochrome *c* oxidase subunit II as well as cytochrome *c* supports a conclusion that increased expression of mitochondrial respiratory chain complex proteins may be a feature of megakaryocyte maturation that is regulated post-transcriptionally. However it is unlikely that *CYCS*-associated thrombocytopenia is related to a requirement for increased levels of respiratory chain complexes in differentiating megakaryocytes. As we have previously noted, Thrombocytopenia Cargeeg subjects have no symptoms indicative of a respiratory chain defect [5], and thrombocytopenia is not a feature of mitochondrial diseases [46,47]. The molecular basis of *CYCS*-associated thrombocytopenia therefore requires further investigation.

## Supporting information

S1 Fig PMA induces differentiation of SET-2 cells. SET-2 cells were plated at 2 × 10^5^ cells/well in a 24-well plate and treated with or without 10 nM PMA. **A**. Cell growth analysis using the IncuCyte^®^ Live-Cell Analysis System. % Confluency of cells treated with (Δ) or without (□) PMA. **B**. Percentage of cells expressing CD61 (white bars) or CD61 and CD41a (black bars) as determined by flow cytometry. **C**. Percentage of platelet-like particles expressing CD61 and/or CD41a. Data are presented as mean ± SD (n = 3).
(PDF)

S2 Fig PMA induces differentiation of MEG-01 cells. MEG-01 cells were plated at 2 × 10^5^ cells/well in a 24-well plate and treated with or without 5 nM PMA. Percentage of cells expressing CD61 as determined by flow cytometry. Data are presented as mean ± SD (n = 3).
(PDF)

S3 Fig Effect of PMA on cytochrome c mRNA expression. SET-2 cells were treated with 10 nM PMA for 0–72 h and relative cytochrome c mRNA was determined by qPCR. Data are presented as mean ± SD (n = 3). *P<0.05 compared to 0 h by one sample *t* test (GraphPad QuickCalcs).
(PDF)
