# Supplementary figures and images for: Differentiation and cell density upregulate cytochrome c levels in megakaryoblastic cell lines: Implications for analysis of CYCS-associated thrombocytopenia
Source: PLoS One. 2017 Dec 29;12(12):e0190433. doi: 10.1371/journal.pone.0190433 (PMC5747465; doi:10.1371/journal.pone.0190433)

A

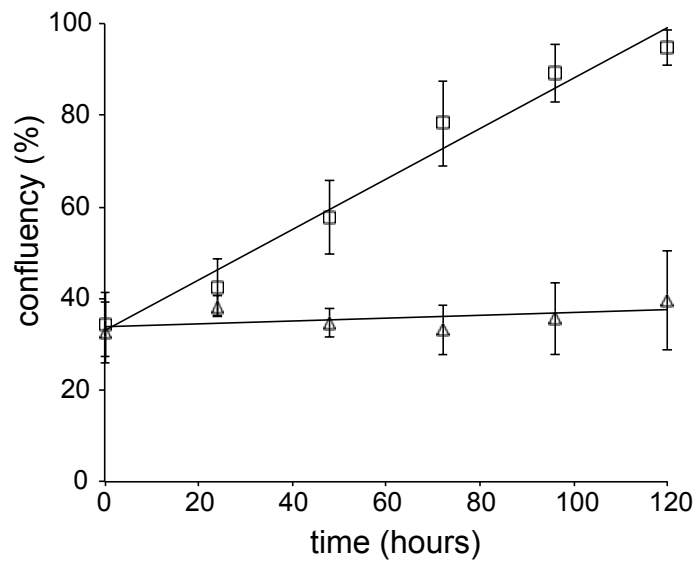

B

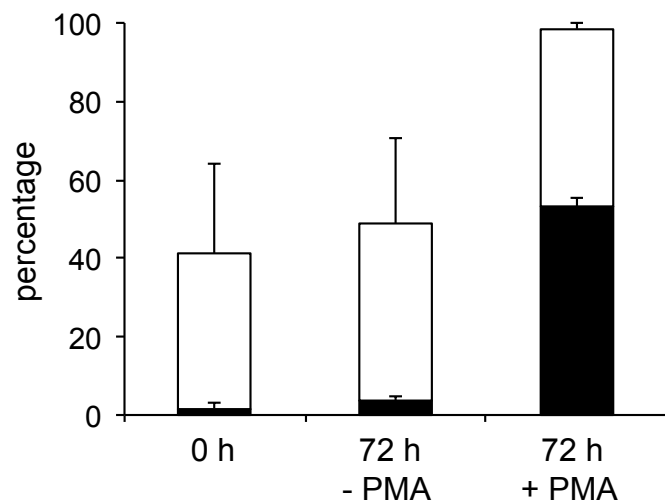

C

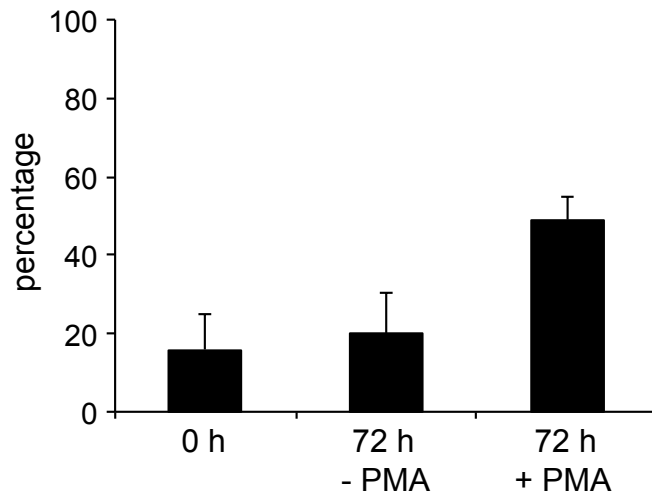

Supplement: S1 Fig — SET-2 cells were plated at 2 × 10^5^ cells/well in a 24-well plate and treated with or without 10 nM PMA. A. Cell growth analysis using the IncuCyte® Live-Cell Analysis System. % Confluency of cells treated with (Δ) or without (□) PMA. B. Percentage of cells expressing CD61 (white bars) or CD61 and CD41a (black bars) as determined by flow cytometry. C. Percentage of platelet-like particles expressing CD61 and/or CD41a. Data are presented as mean ± SD (n = 3). (PDF) [file pone.0190433.s001.pdf]

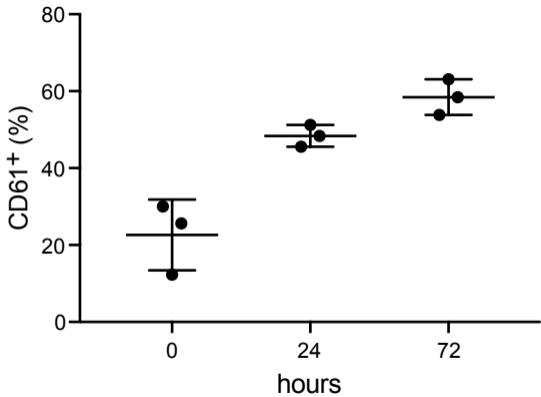

Supplement: S2 Fig — MEG-01 cells were plated at 2 × 10^5^ cells/well in a 24-well plate and treated with or without 5 nM PMA. Percentage of cells expressing CD61 as determined by flow cytometry. Data are presented as mean ± SD (n = 3). (PDF) [file pone.0190433.s002.pdf]

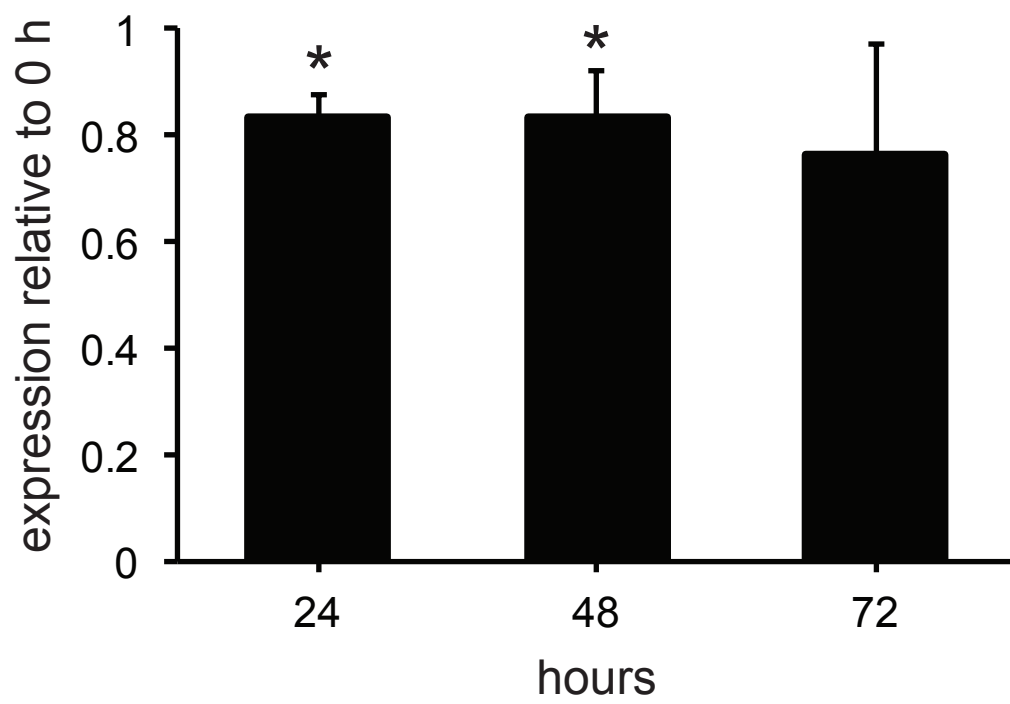

Supplement: S3 Fig — SET-2 cells were treated with 10 nM PMA for 0–72 h and relative cytochrome c mRNA was determined by qPCR. Data are presented as mean ± SD (n = 3). *P<0.05 compared to 0 h by one sample t test (GraphPad QuickCalcs). (PDF) [file pone.0190433.s003.pdf]
